# 19455 The impact of long-term construction on the health of older adults in New York City™s Chinatown

**DOI:** 10.1017/cts.2021.614

**Published:** 2021-03-30

**Authors:** Janet Pan, Yi-Ling Tan, Jennifer Wong, Stella Chong, Isabel Ching, Jan Lee, Judith Zelikoff, Simona Kwon

**Affiliations:** 1 Department of Population Health, NYU Grossman School of Medicine; 2 Hamilton-Madison House; 3 Chinatown Core Block Association; 4 Department of Environmental Medicine, NYU Grossman School of Medicine

## Abstract

ABSTRACT IMPACT: This poster will demonstrate how a community issue from a CTSI Community Advisory Board member organization initiated a collaborative, community-engaged project to identify priority areas of concern and culturally appropriate mitigation strategies. OBJECTIVES/GOALS: Little is known about the health and psychosocial impact of construction on older adults living near construction sites. We applied a mixed methods approach to identify evidence-based strategies to mitigate community prioritized health and psychosocial concerns related to long-term construction on older adults in NYC’s Manhattan Chinatown. METHODS/STUDY POPULATION: In Chinatown, where approximately 20% of its residents are seniors, many are poor, have a disability, and experience ambulatory difficulties. We used a mixed methods approach including: 1) a high level scoping review of the published literature on the health impact of long-term construction for older adults; 2) key informant interviews with stakeholders; and 3) a two-part community-engaged modified Delphi process to identify priority topic areas related to construction and older adults and evidence-informed, culturally-relevant mitigation strategies. Using priority areas identified through the modified Delphi process, we conducted a literature review on the health and psychosocial impact of construction on older adults. RESULTS/ANTICIPATED RESULTS: We identified five priority topics: construction site emissions; noise; outdoor nocturnal lighting; neighborhood changes; and relocation. Long-term construction is associated with environmental and psychosocial consequences with greater negative impacts on vulnerable populations. Current NYC mitigation policies are based on general population and need revisions to consider impacts for the most vulnerable, e.g. older adults and children, to mitigate adverse health outcomes. Findings were shared with City Council members and resulted in enacting specific recommended mitigation strategies, e.g. double paned windows, etc. Seniors are highly susceptible to the effects of air pollution, noise, and environmental changes, with exposure associated with higher morbidity, mortality, and social isolation. DISCUSSION/SIGNIFICANCE OF FINDINGS: Long-term construction may pose serious health implications for seniors residing near construction sites. Standards and guidelines for the general population may not protect them. Community-driven coalitions, like community-academic partnerships, can successfully advance community priorities and inform strategies to protect the elderly.

